# Perspectives of Patients With Early Psychosis on the Use of an App in Acceptance and Commitment Therapy: A Qualitative Study

**DOI:** 10.1111/eip.70073

**Published:** 2025-08-05

**Authors:** Jara Bouws, Lotte Uyttebroek, Joanne R. Beames, Mariken de Koning, Frederike Schirmbeck, An Henrard, Ulrich Reininghaus, Lieuwe de Haan, Inez Myin‐Germeys

**Affiliations:** ^1^ Arkin, Centre for Mental Health Care Amsterdam the Netherlands; ^2^ Department of Psychiatry, Amsterdam UMC University of Amsterdam Amsterdam the Netherlands; ^3^ Department of Neurosciences, Psychiatry Research Group, Centre for Contextual Psychiatry KU Leuven Leuven Belgium; ^4^ Department of Public Mental Health, Central Institute of Mental Health, Medical Faculty Mannheim Heidelberg University Mannheim Germany; ^5^ Z.ORG KUL Academic Psychiatric Centre KULeuven Kortenberg Belgium

**Keywords:** acceptance and commitment therapy, ecological momentary assessment, first episode psychosis, mental health, qualitative study, smartphone app

## Abstract

**Introduction:**

Individuals with early psychosis received Acceptance and Commitment Therapy in daily life (ACT‐DL), consisting of 8 face‐to‐face sessions and use of a mobile app at home, as part of a randomised controlled trial (INTERACT). Those receiving ACT‐DL showed improvement in negative symptoms and global functioning compared to the control condition. The current study qualitatively explores patients' perspectives on the ACT‐DL app and perceived areas for improvement.

**Methods:**

The ACT‐DL app prompted individuals randomly multiple times a day between therapy sessions to complete questionnaires (Ecological Momentary Assessments, EMA) and ACT metaphors or exercises (Ecological Momentary Interventions, EMI). User experiences with the ACT‐DL app were explored in 17 semi‐structured interviews within 6 months after the intervention and analysed using template thematic analysis.

**Results:**

Three themes were formed: 1. App functionalities and usability; consisting mainly of perceived practical obstacles. 2. Additional value of the app; on how the EMAs raised levels of awareness for feelings, thoughts, and behaviour, and the positive evaluation of the ACT exercises in the EMI part of the app. 3. Improving applicability and effect of the ACT‐DL app; with practical feedback from participants.

**Conclusions:**

Individuals with early psychosis were generally positive about the effects of the ACT‐DL app, attributing benefits to increased awareness via EMAs and to the ACT exercises. However, they experienced difficulties using the app during work and social activities. Participants provided valuable suggestions to improve the app's effectiveness and applicability.

**Abbreviations**
ACT: acceptance and commitment therapy
ACT‐DL: acceptance and commitment therapy in daily life
CHR‐P: clinical high risk for psychosis
EMA: ecological momentary assessment
EMA/I: ecological momentary assessment and intervention
EMI: ecological momentary intervention
ESM: experience sampling method
FEP: first episode psychosis

## 1 Introduction

The rapid growth of digital technology offers new opportunities for mental health care. Ecological Momentary Assessment (EMA) (Shiffman et al. 2008; Stone and Shiffman 1994), also known as the Experience Sampling Method (ESM) (Csikszentmihalyi and Larson 1987; Myin‐Germeys and Kuppens 2022), is a research methodology involving recurrent real‐time self‐monitoring of emotions, thoughts, and behaviours in real‐world contexts, commonly delivered through smartphone apps. EMA offers valuable information for clinical treatment and research by providing insights into an individual's daily inner world, enabling the identification of patterns in emotional experiences, symptoms, and their determinants across diverse contexts (Myin‐Germeys et al. 2009, 2018; Trull and Ebner‐Priemer 2009; van Os et al. 2017). Utilising EMA in therapy minimises retrospective biases by assessing individuals' current feelings instead of relying on recollections about the past period (Solhan et al. 2009). Ecological Momentary Intervention (EMI) refers to therapeutic strategies integrated into daily life, often utilising EMA, to promote adaptive behaviour and coping mechanisms. EMI involves prompts with reminders, psychoeducation, or psychotherapeutic exercises (Heron and Smyth 2010; Myin‐Germeys et al. 2016).

Individuals with early psychosis, including individuals at Clinical High Risk for Psychosis (CHR‐P) or with First Episode Psychosis (FEP), commonly experience not only (attenuated) psychotic symptoms but also impaired cognitive and global functioning, comorbid mental disorders, and lower quality of life (Fusar‐Poli et al. 2017, 2020; McGorry et al. 2006; Yung et al. 2005). Tackling these additional issues that affect everyday life is challenging (Mei et al. 2021). Combining EMA and EMI (EMA/I) with face‐to‐face psychotherapy for early psychosis holds promise in this respect, as it could facilitate the application and integration of acquired skills from therapy into daily routines (Bell and Alvarez‐Jimenez 2019; Valentine et al. 2020). A systematic review (Bell et al. 2017) identified nine EMA and/or EMI interventions in psychosis treatment and showed overall feasibility, acceptability, and positive clinical outcomes. However, the interventions varied considerably in terms of their objectives (e.g., relapse monitoring, medication adherence, personal goal attainment, symptom management or coping), delivery mode of EMA and/or EMI (e.g., SMS, telephone, sms or telephone with a web‐based management system, smartphone application, PDA), duration (ranging from 4 to 104 weeks), and study design (e.g., RCT, single‐arm or pre–post trial, pre–post trial with historical control). This heterogeneity, combined with a lack of qualitative insights into participants' perspectives on using EMA/EMIs, limits our understanding of the (subjective) effects in individuals with psychosis.

Acceptance and Commitment Therapy (ACT) (Hayes et al. 2006) is a transdiagnostic therapy (A‐Tjak et al. 2015) that aims to enhance psychological flexibility: being aware and accepting of unpleasant thoughts and feelings, whilst focusing on the present moment and engaging in value‐based actions (Hayes et al. 2006). ACT has been proven to be feasible and acceptable for individuals with (early) psychosis (Bouws et al. 2024; Gaudiano and Herbert 2006; Johns et al. 2016; Morris et al. 2024). Furthermore, ACT does not only show promising results in the treatment of psychotic disorders but might also improve treatment adherence for people with psychotic disorders (Gaudiano and Busch 2013).

Acceptance and Commitment Therapy in Daily Life (ACT‐DL) is an innovative blended care intervention, combining eight face‐to‐face ACT sessions with a smartphone‐based EMA/I intervention to be used at home, the ACT‐DL app. The efficacy of ACT‐DL was investigated in the INTERACT trial (Myin‐Germeys et al. 2022; Reininghaus et al. 2019), a randomised controlled trial that compared treatment as usual (TAU) with ACT‐DL in addition to TAU for individuals with CHR‐P and FEP. ACT‐DL is meant to promote the application of ACT principles in the real world and has proven to yield significant improvements in momentary distress, global functioning, and negative symptoms (Myin‐Germeys et al. 2022). However, whilst ACT‐DL demonstrates feasibility in terms of treatment adherence, usefulness, and acceptability, it is to some extent burdensome, particularly regarding the length and frequency of the EMA (van Aubel et al. 2020, 2024). Existing feasibility data on EMA/I for early psychosis (Bell et al. 2017), on ACT‐DL for early psychosis (Vaessen et al. 2019) and on EMA/I in general (Yung et al. 2005), primarily rely on quantitative evidence and lack detailed insights into patients' experiences with EMA/I alongside face‐to‐face therapy. This qualitative study aims to explore patients' perspectives on the ACT‐DL app in general and to identify potential areas for improvement. These findings could contribute to refining the ACT‐DL and enhancing other blended care interventions incorporating EMA/I for individuals in the early stages of psychosis.

## 2 Materials and Methods

### 2.1 Design and Participants

Participants for the current qualitative study were recruited from participants of the INTERACT trial who were randomised to the ACT‐DL condition. The INTERACT participants were included in Belgium and the Netherlands based on the following criteria: age 15–65, meeting the criteria for CHR‐P or FEP (onset within 3 years), sufficiently speaking the Dutch language and having the ability to give written informed consent. Participants randomised into the experimental condition received 8 weeks of ACT‐DL intervention in addition to treatment as usual at their mental health care facility. Participants could receive a total of 145 euro for attending study assessments, but 15 euro was retracted for missing an entire week of EMA/I prompts. The trial protocol and main results are documented elsewhere (Myin‐Germeys et al. 2022; Reininghaus et al. 2019; Vaessen et al. 2019; van Aubel et al. 2020), but for the current study it is relevant to mention that participants receiving the ACT‐DL intervention had an average of 13 (SD 8.7) interactions per week with the ACT‐DL app, of which 6 per week (from a total of 24 in the 3 days after therapy) were responses to EMA questionnaires. From the 71 participants in the ACT‐DL condition, 46 completed a debriefing questionnaire on acceptability of the intervention. 89% reported that the EMA/EMI component was (to some extent) useful, 91% reported that it helped them to apply ACT in daily life, and 85% reported that it increased emotional awareness. Further, 91% indicated that the number of prompts was to some extent burdensome and 91% reported that the number of items in a questionnaire was to some extent burdensome (van Aubel et al. 2024).

Recruitment for the qualitative study took place between June 2018 and September 2019, using convenience sampling. A research coordinator approached all ACT‐DL arm participants who were within 6 months post ACT‐DL treatment (*n* = 30) in the recruitment period, regardless of intervention completion, inviting them for a semi‐structured interview, as part of the INTERACT trial. An additional 10 euro gift voucher was received when participating in the qualitative part of the study.

### 2.2 Intervention

**FIGURE 1:**
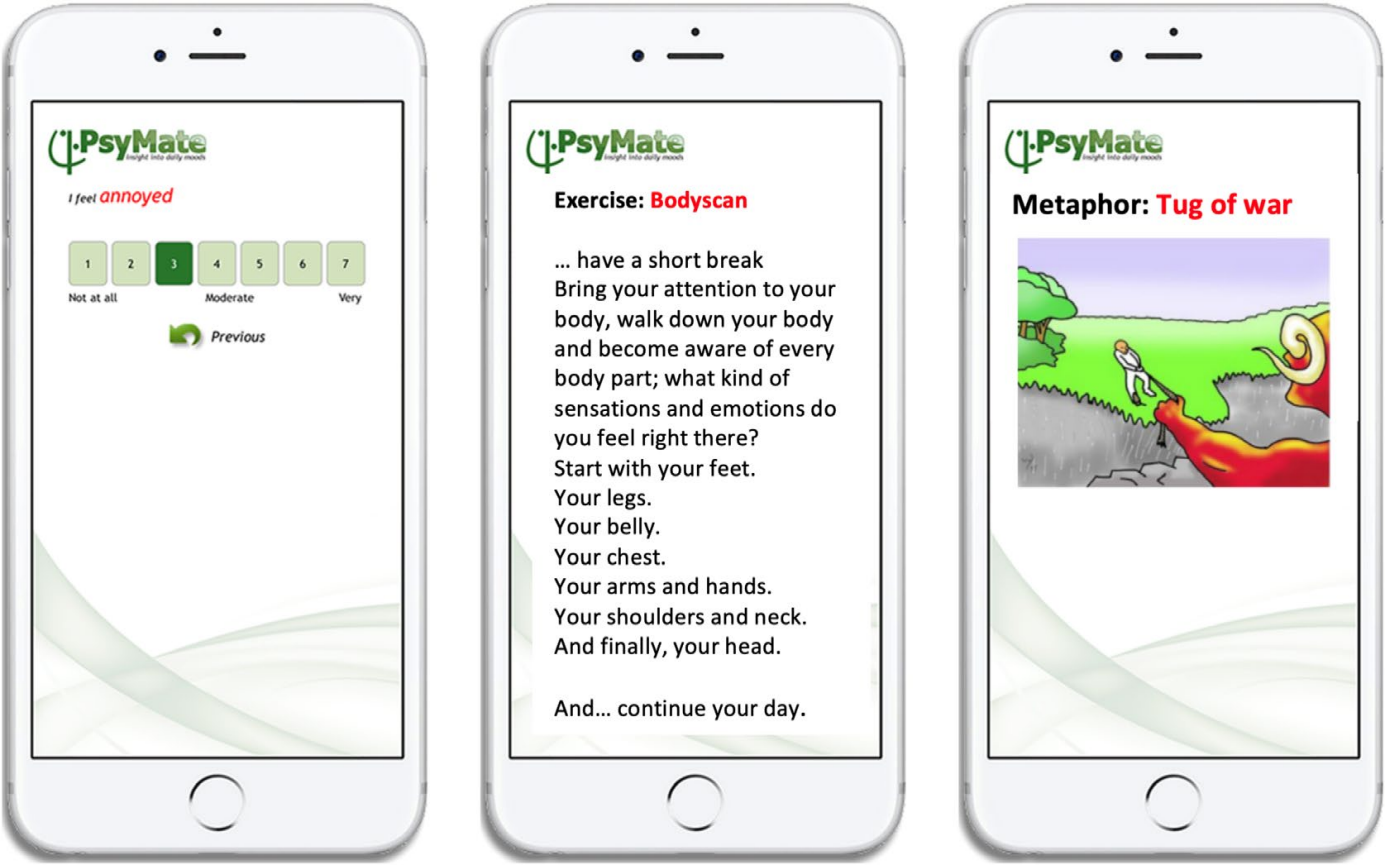
Example of EMA question, EMI ACT mindfulness exercise, and EMI ACT‐metaphor.

The Acceptance and Commitment Therapy in Daily Life (ACT‐DL) intervention consisted of eight face‐to‐face ACT therapy sessions with a trained ACT clinician, combined with the use of the ACT‐DL app: an ACT‐based EMA/I administered through a smartphone app (PsyMate, www.psymate.eu). This was provided on a study mobile phone, because at the time, developers of PsyMate could not guarantee compatibility across different mobile operating systems. The first session provided psychoeducation on early psychosis; the following six sessions focused on the main components of ACT: creative hopelessness, acceptance, defusion, the self and mindfulness, values, and commitment (Hayes et al. 2006; Reininghaus et al. 2019). The final session aimed to discuss and integrate what was previously learned. Further details regarding the content of the sessions have been reported elsewhere (Vaessen et al. 2019). The ACT‐DL app was used for three consecutive days after each therapy session to stimulate the transfer of ACT skills in daily life and to enhance mindfulness. Participants were randomly prompted eight times daily to complete an EMA questionnaire (“beeps”) comprising 17 questions on mood, symptoms, company, physical well‐being, current activity, and substance use, see Figure 1. Furthermore, participants received a morning and evening EMA questionnaire, focusing respectively on sleep quality and on daily reflections. Responding to each beep was time‐limited to 15 min. The EMI component, following each questionnaire, presented either a written ACT exercise or an ACT metaphor through the ACT‐DL app (ratio 50:50), which were based on what was learned in the previous session. See Figure 1 for examples of the ACT‐DL app screen and Supplement 1 for an example of how an ACT metaphor was explained in session and/or workbook. When an ACT exercise was presented, participants were given the option to choose between an exercise related to the ACT principle covered in the previous week's face‐to‐face session or a mindfulness exercise. Additionally, participants had the option to independently listen to MP3 audio files containing ACT mindfulness exercises via the media player on the study phone. Each session began with a brief evaluation of how the past week had gone, including questions about whether the app had been used successfully and whether it had functioned properly.

### 2.3 Interviews

A semi‐structured interview guide was developed by JB and AH. The interviews explored participants' experiences with Acceptance and Commitment Therapy and the associated ACT‐DL app received in the INTERACT trial. The interview guide is available on Open Science Framework (https://osf.io/5wndk/files/osfstorage/632f58bb18f4581022428b2a). Findings on experiences with the ACT sessions are described elsewhere (Bouws et al. 2024).

The interviews started with collecting demographic information and explored three main topics regarding the ACT‐DL app: (1) participants' experiences with the app, (2) the potential added value of the ACT‐DL app in treatment and integration of ACT into daily life, and (3) suggestions for improvement or alterations of the app. Questions were open‐ended and participants were encouraged to elaborate on aspects they considered significant or valuable. Interviews were conducted by JB and AH, ranging from 34 to 84 min (average duration 48 min) and were audio recorded.

JB is a psychiatrist and PhD candidate at the University Medical Centre and Arkin, a mental health care institution in Amsterdam, the Netherlands. She has several years of experience working with individuals with (early) psychotic disorders, and her research focuses on therapeutic interventions for early psychosis. At the time of the interviews, she had limited prior knowledge of Acceptance and Commitment Therapy (ACT), which may have been both an advantage—allowing her to approach interviews with an open, unbiased, and non‐suggestive stance—and a disadvantage, as it might have led to overlooking ACT‐specific content. AH is a clinical psychologist based in Kortenberg, Belgium, who primarily works with inpatients diagnosed with schizophrenia. She had received training in ACT at the time of the interviews, which allowed her to recognise and elaborate on ACT specific content but could have biassed her questioning.

### 2.4 Analysis

Sample characteristics were reported using descriptive summary statistics. Interviews were transcribed verbatim by JB and AH and imported into NVivo for coding. Template analysis (Brooks et al. 2015; King 2025), a form of codebook thematic analysis (Braun and Clarke 2022), was employed for qualitative data analysis. JB and LU created a set of general a priori themes. After independently coding three interviews, JB and LU discussed the results to create the initial coding template. JB coded the rest of the interviews top‐down using this coding template. Bi‐weekly meetings between JB and LU were held to interpret the data and adjust the coding template.

Regarding trustworthiness criteria for qualitative research (Korstjens and Moser 2018; Nowell et al. 2017); JB and LU worked extensively and thoroughly on this research. Furthermore, they discussed and kept records of research steps, personal assumptions, and reflections. They consulted JRB and MdK on several occasions for peer debriefing to discuss theme development and reduce researcher bias.

LU is a psychologist and PhD candidate at KU Leuven, Belgium. Her research focuses on the use of Experience Sampling Methodology (ESM) across a range of psychiatric disorders. JRB is a psychologist and associate researcher at KU Leuven, with broad research interests that include, amongst other topics, adolescent mental health. MdK is a psychiatrist and senior researcher at Arkin in Amsterdam, the Netherlands, with extensive experience in treating individuals across the psychosis spectrum and in conducting qualitative research with this population.

All aforementioned authors (JB, AH, LU, MdK, and JRB) were not involved in the quantitative component of the INTERACT study. However, they inevitably did hold certain expectations regarding potential positive and negative effects of ACT and ACT‐DL for early psychosis. Whilst the first author (JB) did not hold any hopes for a positive or negative evaluation of the ACT‐DL app, she did, after reviewing the INTERACT study protocol, anticipate that participants might express concerns about the perceived burden of using the ACT‐DL app.

## 3 Results

### 3.1 Sample

Of the 30 eligible participants for the qualitative study, 6 (all Belgian) could not be reached and 5 (all Dutch) did not want to participate, resulting in a total of 19 participants (12 Dutch and 7 Belgian) who were interviewed. Two interviews were lost due to technical issues.

Of all participants, 7 FEP participants had been admitted to a psychiatric hospital for psychosis before or during the INTERACT study. Of the 6 CHR‐P participants, 4 had continued work and/or studies despite their symptoms, 1 was just restarting work at the time of the interview, and 1 participant reported limited functioning at school or study. All 11 FEP participants used to work and/or study in the past but had stopped (temporarily) because of psychotic symptoms; 5 had not returned to work or studies at the time of the interview (Table 1).

**TABLE 1:** Participant demographic characteristics.

Characteristics	Participants, *n* (%)
Total	17 (100)
Age	
21–30	10 (59)
31–40	7 (41)
Sex	
Female	12 (71)
Male	5 (29)
Country	
The Netherlands	12 (71)
Belgium	5 (29)
Diagnosis	
CHR‐P	6 (35)
FEP	11 (65)
Employment status	
None	4 (23)
On sick leave	3 (18)
Working	7 (41)
Studying	2 (12)
Working and studying	1 (6)

### 3.2 Analytic Process

The interviews produced rich, in‐depth, and often lengthy descriptions of participants' experiences. Themes were a priori expected to show similarity with the semi‐structured interviews topics about the app, thus consisting of barriers/negative experiences with the ACT‐DL app, facilitators/positive experiences with the ACT‐DL app, and suggestions for improvement. Coding and discussion of the initial three interviews revealed challenges in capturing the ambivalence and contradictory nature of experiences and opinions within these a priori themes. Subsequently, an adjustment was made to the initial coding template, resulting in five themes for the initial coding template: app design and protocol, applicability of ACT‐DL in life, integration of ACT‐DL app and therapy, perceived effectiveness of the app, and suggestions for improvement. During the coding process, themes that had substantive overlap were merged, and theme names were made more specific, resulting in the following three themes: App Functionalities and Usability, Additional Value of the App and Improving Applicability and Effect.

Counting and reporting the number of codes that had similar meanings aimed to identify patterns and facilitate future adjustments to the app (Sandelowski 2001).

### 3.3 Theme 1: App Functionalities and Usability

The theme app functionalities and usability captures reflections that participants shared about their user interactions with the ACT‐DL app. In this context, participants mainly focused on the EMA (questionnaires) component in the interviews. We defined two sub‐themes: (1) number and repetition of beeps and questions and (2) interference with work and social life. Illustrative quotes on the sub‐themes are presented in Table 2.

#### 3.3.1 Number and Repetition of Beeps and Questions

Participants had diverse opinions on the number of beeps and the length of the questionnaires in the EMA protocol. Three participants considered the number of beeps appropriate, four deemed it excessive, and five expressed mixed feelings. Regarding the questions per beep, five participants found the quantity satisfactory, whilst four deemed it too many. When the duration of answering a questionnaire was made specific, it ranged between 30 s and 5 min. Nine participants expressed discomfort with the repetition and fixed order of questions at each beep, stating that it negatively influenced the validity of their responses: some participants reported reduced focus on questions or providing answers without thoughtful consideration. One participant even mentioned changing a negative response about hallucinations to a positive one due to the repetitive nature of the question, fearing she may have answered incorrectly initially.

#### 3.3.2 Interference With Work and Social Life

Thirteen participants reported that integrating the ACT‐DL app into their daily routines was challenging. Eight participants indicated that complying with the ACT‐DL app at work or college had been or would have been impossible; another two participants refrained from bringing it to college or work because they anticipated being unable to comply. The reasons cited include beep frequency, time required to complete questionnaires, and inconvenient timing of the beeps. Six participants encountered challenges using the ACT‐DL app in social situations, such as dining out or during family holidays, mentioning feelings of intrusiveness or embarrassment.

**TABLE 2:** Illustrative quotes from theme 1.

Theme	Participant	Quote^a^
Number and repetition of beeps and questions	1	But évery hour, this annoying BEEP […] Every hour, 10 h in a row.
	6	It really depended on what I did on a day. Some days I found it [amount of beeps] really annoying. But on other moments, when I had a hard time in my head, and could not stop thinking, I mean when I was in conflict with myself, the app could really pull me out of it. So sometimes it was really nice that it beeped. It depended, but mostly I benefited from it.
	14	It was a lot of questions for me. And too many beeps. […] And, after a while, all the time the same questions, this was also unpleasant.
	2	The same questions come every time in the same order, so at a certain point you have a standard answer, and you don't read the question anymore.
	4	Some questions about symptoms that I did not recognise were repeated so often, that after a couple of weeks I filled in that I did recognise it. I thought that maybe I was crazy that I did not notice I had this symptom.
Interference with work and social life	10	When I had the phone [ACT‐DL app] the first week, I took it to work and told colleagues that it was a little test. This was fine. But later, in between the sessions, I could not use the phone [ACT‐DL app] at work, it distracted me. It also would have raised questions at work if this “test” took 2 months.
	8	At the time ACT started, I was just starting up with a few customers at home [hairdressing], which was really important for me […]. But I couldn't tell a customer: sorry, I have to answer some beeps for my psychosis […] And I felt really bad, a failure, and thought the therapists and research group would think I did not take it seriously.
	13	Because I was not working, it was easy and quite nice to do it [the beeps], but if I would have had a job at the time, it would have been hard and annoying.
	17	I put it [ACT‐DL app] on mute during class, so I missed many beeps, but I also did not dare answering the questions in class.
	1	Yes, I did everything [EMA/I], but I told them: look, when I go out for dinner, I won't bring this thing [ACT‐DL app], or when I am playing a game with my son.

*Note:* App functionalities and usability.

^a^ Quotes were translated from Dutch/Flemish and slightly adjusted to enhance readability.

### 3.4 Theme 2: Additional Value of the App

The theme additional value of the app describes the perceived psychological benefits of the ACT‐DL app as well as its potential role in facilitating the integration of ACT skills into daily life. We categorised three sub‐themes: (1) EMA‐raised awareness, (2) exercises and metaphors, and 3) no additional value of the app. See Table 3 for illustrative quotes.

#### 3.4.1 EMA Raised Awareness

The EMA prompts with questionnaires created a higher level of awareness, according to eight participants. They reported being more aware of how they felt during or after filling out the questionnaires. They also noticed being more aware of how their day was going or what activities they had (not) done up to that point, which could work as an incentive to become more active or result in a more positive reflection on their activities up to that point. One participant mentioned that this awareness, caused by the EMA prompts, grounded her and prevented her from getting lost in her symptoms. Another participant said that the EMA questionnaires raised awareness without being judgmental, which was a drawback of other available apps such as mindfulness apps.

#### 3.4.2 Exercises and Metaphors

Nine participants appreciated the link between the sessions and the exercises (EMI part) in the ACT‐DL app. In contrast, metaphors, the other EMI part of the ACT‐DL app, were not often mentioned.

One participant expressed a preference for the end phase of the treatment period, noting that all exercises and metaphors were available in the app at that stage. Three participants explicitly mentioned that the exercises separately provided via the media player on the study phone were more helpful than those provided via the ACT‐DL app. The perceived advantages of the media player were that these were audio files instead of text and that all exercises were available at once, meaning that there was a greater selection to choose from (as opposed to being added each week). One participant experienced a weak connection between the sessions and the exercises, and when prompted with metaphors from the last session, she had forgotten how to interpret them.

#### 3.4.3 No Additional Value of the App

One participant thought the questionnaires were for the therapist to monitor the participant and had no purpose for the participant. Another participant did not see an added value of the ACT‐DL app and got annoyed with filling in numbers; it felt like a waste of time. A third participant explained that the questionnaires in the ACT‐app forced her to think a lot, instead of helping her to be in the moment and let go of thoughts, thus it had an opposite, negative effect.

**TABLE 3:** Illustrative quotes from theme 2.

Theme	Participant	Quote^a^
EMA raised awareness	16	Every beep I had a moment to realise: what am I doing, how do I feel? That worked, because usually I am so busy I am not aware of these things. Things like: “I am actually very tired”, or “I really did not like that”, or “I had a good time”.
	4	It worked as a check‐in a couple of times per day (questions like: what are you doing?, who are you with?). Sometimes I realised I answered the same last 3 beeps and thought “maybe it is time to get up and do something” or “maybe it is time to take a break”. […] It also raised a positive awareness, or a feeling of gratitude, when I was having a good day.
	6	Every time I had to answer those questions, I felt like I was getting more aware, and I was really doing something. And when I did not have the phone [ACT‐DL] anymore I did not completely fall back, but symptoms did emerge again. The app grounded me.
	8	And I, mainly because of the phone [ACT‐DL app], became more aware about what influenced my mood, positive and negative.
Exercises and metaphors	13	It worked really well, when we were able to choose exercises in the app, and the app reminded you that they were available. Without the phone [ACT‐DL app] you are much less actively involved with it.
	3	Yes, the app connected with sessions, because the metaphors reappeared in the app, to check if I got it. […] So I got questions and then a metaphor, just like the one in my workbook and then I could practise it.
	15	After each session, the content of the app changed, it focused on what we talked about in the session. I cannot recall all of them, but for example, it was about how you could see your thoughts as clouds and you could get more control over them.
No additional value of the app	14	For me the app did not help integrating therapy, it had an opposite effect. I got confronted with my mind again, and maybe also with my illness. I think living in the now is more interesting, to train that, like mindfulness, and not all those questions.
	7	The purpose of the [ACT‐DL] app is for them to monitor how someone is doing in real‐life; this is more reliable than a retrospective questionnaire. […]. I saw it like tests, like they wanted to constantly check your mood.

*Note:* Additional value of the app.

^a^ Quotes were translated from Dutch/Flemish and slightly adjusted to enhance readability.

### 3.5 Theme 3: Improving Applicability and Effect

This final theme covers elements that, according to participants, could be improved in the ACT‐DL app. Suggestions were practical, mostly protocol‐related, and revolved around two sub‐themes: (1) improving applicability in daily routines and (2) improving therapeutic effect. The second sub‐theme was divided into three lower sub‐themes: (a) longer period of use, (b) deliverance of metaphors and emphasis on exercises, and (c) feedback on EMA. See Table 4 for illustrative quotes on theme 3.

#### 3.5.1 Improving Applicability in Daily Routines

Five participants suggested making the ACT‐DL app available for download on participants' own mobile devices. The perceived advantages of having the ACT‐DL on personal devices included increased accessibility, compliance, and unobtrusiveness (i.e., less attention or questions from other people about the app).

Five participants suggested improving the applicability in daily routines by lengthening or removing the time interval in which participants could respond to a beep, which was expected to increase the amount of completed questionnaires. Three participants suggested reducing the beep frequency, one participant suggested reducing the number of questions, and one participant suggested creating an option for users to choose how many beeps to complete each day.

#### 3.5.2 Improving Therapeutic Effect

##### 3.5.2.1 Longer Period of Use

Eight participants suggested changing the period during which the ACT‐DL app was allowed or intended to be used. Seven of these participants advocated for lasting availability of the ACT‐DL app after ending the ACT treatment, to prevent a loss of recently acquired skills and to promote a longer‐lasting effect. One of these participants specifically mentioned a desire to continue receiving EMA questionnaires (but only once a day), whilst four participants expressed a wish for continued access to specifically the ACT‐ and mindfulness exercises after completing ACT therapy. Additionally, one participant recommended making the ACT‐DL app available to participants immediately after intake, as it could help whilst waiting for therapy.

##### 3.5.2.2 Deliverance of Metaphors and Emphasis on Exercises

Four participants recommended placing more emphasis on doing ACT exercises in the ACT‐DL app, as they found them helpful and enjoyable to do at home. Two of these participants suggested incorporating the ACT audio exercises from the media player on the study phone into the ACT‐DL app. Additionally, three participants advised making all exercises available anytime instead of adding a couple after each session. Two participants mentioned difficulty in remembering the exact meaning of a metaphor when prompted in the ACT‐DL app, as they were only explained in text; they suggested adding corresponding metaphor pictures, as they had seen in the sessions, to the ACT‐DL app.

##### 3.5.2.3 Feedback on EMA

Six participants suggested a form of feedback or integration of their responses to EMA questions in ACT therapy sessions or within the ACT‐DL app itself. They expressed a desire to discuss their EMA answers in ACT sessions or to be provided with a visual overview of their answers in the ACT‐DL app or during face‐to‐face sessions. The advantage, suggested by participants, of providing feedback on the course of personal daily‐life experiences was that it could enhance the therapeutic effectiveness of the ACT‐DL app.

**TABLE 4:** Illustrative quotes from theme 3.

Sub‐theme	Subtheme	Participant	Quote^a^
Improving applicability in daily routines		11	I often forgot to bring the phone [ACT‐DL app] on social occasions. And if I did not forget it, I did not always answer the beeps. It felt strange to get this thing, I mean, it depends on the other person you are with, if you want to explain it […]. Also, at work the use of a second phone would have raised questions, so it would be easier if the beeps were on your own phone, this would make it more private.
		7	It would help to have it on your own phone, so you wouldn't have to bring two devices all the time.
		9	Many times, because I worked an evening shift, I could not do the evening questionnaire. It would be better if I could do the evening questionnaire at the time that I actually go to bed.
		8	It would have worked better for me if questionnaires would be available all day. You tell me to complete 5 questionnaires a day and I choose my own moments.
		1	Instead of 10 times a day, a better frequency would be about 4 or 5 beeps, plus morning‐ and evening questionnaire.
		10	I mean, it takes about 5–6 min per beep, this is long when you are at work. I think the frequency would be doable‐about 5 times during a workday‐if the questionnaires would have been shorter.
Improving therapeutic effect	Longer period of use	12	I asked if I could use the app after ACT treatment, in a period I was not doing well, but this was impossible. […] I would have liked to be able to keep doing the exercises on the app after treatment.
		13	I would have put the app on my own phone after therapy, so you could keep working on it. It would be a pity if what you learned would get lost over time. […] If you would still have the app, with all exercises available, you would keep using it. […] Not with the beeps, but the exercises available when you want and maybe sometimes a reminder.
		8	Now, after 8 weeks of working on it, it suddenly finished. And look, if I do something like this, I do it to really learn something that could stay with me for a long time. […] Longer use of the app could help. Maybe just once at the end of the day, some questions like “How was your day?”, “What made you (un) happy?”. Or just once a week, a couple of questions, for example “Did you do something for you this week?” or a reminder of a metaphor.
	Emphasis on exercises and deliverance of metaphors	12	The exercises on the app, maybe there could have been more. I liked the fact that you could do an exercise from the last session, and you could choose from an increasing amount of exercises in the app.
		10	I preferred the MP3 files that we got, with audible meditation sessions, over the app with the exercises only in text. […] I found meditation exercises online, but I keep wondering if these are as good as the [ACT‐DL] app. I asked to keep using the MP3 exercises after therapy but that was impossible. […] It's like, you make an app, put it in Play Store, let people pay for it, but make it free for the people who had sessions in mental health care settings. […] Also, it was advised to do the exercises more than twice a week [during treatment], nonsense, the advice should be to do them every day! Would be so nice, every evening before you go to sleep.
		15	I think the focus of the app was on the questions and less on the exercises, maybe this should have been the other way around.
		16	When the metaphors were prompted in the app, I sometimes did not remember what they meant. It might be better to add the picture of the metaphor in the app […] Now it only says: think about “man with the stick”, for example.
	Feedback on EMA	11	In the beginning it felt nice because the app felt like someone was keeping an eye on me, so I thought when I felt really bad, that if someone else would know it, I would not be so alone with it. But when nobody gets back to you about it, you feel like nobody received your data.
		12	It would be interesting when the app answers would be sent to your therapist and you can talk in session about your different moods of last week. […] Maybe it would be possible to show graphics on how you evolved on the different questions, maybe with tips and feedback, for example “this is a difficult thing for you, you can work on it like this.”
		2	The long questionnaires created a little more awareness on how you felt, but it would be great if you could see results afterwards. It would have created more awareness about what is right and wrong for me. […] Because now it disappeared, such long questionnaires, so many times a day, and it is not of much use to yourself. […] I would have been more motivated to answer the questionnaires.
		9	For example, you had to answer how enthusiastic, happy or down you felt. It is possible that in the session I think I had a good week, but on the app I expressed many negative emotions. This is very informative for the therapy. I often don't realise how I feel or felt, the app could have added to my awareness.

*Note:* Improving applicability and effect.

^a^ Quotes were translated from Dutch/Flemish and slightly adjusted to enhance readability.

## 4 Discussion

### 4.1 Principal Findings

This qualitative study aimed to explore the experiences of participants with CHR‐P or FEP with the ACT in Daily Life (ACT‐DL) app, a smartphone‐delivered EMA/I. Participants utilised the ACT‐DL app alongside eight face‐to‐face ACT sessions within the INTERACT study, a randomised controlled trial (Myin‐Germeys et al. 2022). Our key findings from template analysis (Brooks et al. 2015; King 2025) of 17 semi‐structured interviews revealed that using the ACT‐DL app heightened awareness across various aspects of participants' lives and facilitated the integration of ACT therapy into daily routines. However, challenges were noted regarding the applicability of the ACT‐DL app within participants' social, study, or work‐related schedules, and some reported adverse effects associated with EMA.

### 4.2 Comparison With Previous Work

Other qualitative studies on the implementation of EMA in the daily life of different patient populations reported congruent outcomes concerning increased (self‐)awareness. Findings from a feasibility and acceptability study focusing on EMA for recently discharged patients with psychotic‐spectrum disorders (Moitra et al. 2017), which included a small qualitative component, indicated that EMA was beneficial for certain participants by enhancing their awareness of symptoms and (un)productive activities. Another qualitative study on ESM, comprising long‐term EMA monitoring, episode alerts and personalised feedback to clinical care for patients with bipolar disorder (Bos et al. 2020), revealed that almost all patients reported increased awareness of (factors influencing their) mood, symptoms and behaviour. A qualitative study on EMA for veterans with non‐suicidal self‐injury (NSSI) reported that numerous participants mentioned the acquisition of self‐knowledge, specifically awareness and insight into their internal experiences (Gromatsky et al. 2022).

Interestingly, a substantial proportion of participants in other qualitative studies (Bos et al. 2020; Gromatsky et al. 2022) experienced the EMA as “reminders” of their symptoms or disease and described this as confrontational and sometimes worsening or triggering symptoms. In our study, this aspect was mentioned by only one of 17 participants. The mainly positive value of increased self‐awareness in our study might be attributed to the hybrid design, where EMA was integrated into the context of ACT face‐to‐face sessions. Blending the use of apps with evidence‐based, i.e., face‐to‐face, therapy has been mentioned as a field worthy of investigation in a meta‐analysis that found insignificant or small pooled effects of stand‐alone smartphone‐based interventions for mental health issues (Weisel et al. 2019). Furthermore, ACT itself may have equipped participants with tools to acknowledge and accept thoughts, feelings, and symptoms, rather than discard them as unwanted (Hayes et al. 2006), thereby inherently aligning well with the heightened awareness fostered by EMA. This synergy between the two interventions has the potential to amplify their impact, as they complement each other in promoting self‐awareness and acceptance.

The EMI segment, particularly the ACT and mindfulness exercises on the ACT‐DL app and/or media player on the study phone, emerged as an important positive factor for our participants. The exercises were perceived as useful and as promoting the integration of ACT principles into daily life. Furthermore, participants suggested placing more emphasis on, and expanding the availability of, the exercises during ACT‐DL. Qualitative literature on ACT‐ or mindfulness exercises for (early) psychosis is, to our knowledge, scarce. However, in one qualitative study on a lifestyle programme‐including mindfulness‐for FEP, participants reported still using mindfulness 6 weeks post‐treatment to stay grounded and to assist in making healthy lifestyle changes (Thompson et al. 2021). Quantitative literature on mindfulness for FEP shows general feasibility and acceptability (Li et al. 2021; von Hardenberg et al. 2022) and reduced negative symptoms in the short term (MacDougall et al. 2024). A mindfulness‐based online social therapy for people with CHR‐P showed promising results in terms of improving social functioning (Alvarez‐Jimenez et al. 2018). The INTERACT trial demonstrated improvements in negative symptoms and global functioning amongst ACT‐DL participants compared to those in the control condition. Based on participants' subjective experiences in the current study, as well as findings from previous research, it appears that the at‐home ACT (mindfulness) exercises may have played an important role in these outcomes. Enhancing the ecological momentary intervention (EMI) by tailoring the type of exercise or metaphor delivered via the ACT‐DL app to participants' responses on ecological momentary assessment (EMA) questionnaires—that is, aligning the interventions more closely with individuals' daily experiences—may further increase its effectiveness.

Our participants suggested that the therapeutic effect of the ACT‐DL app could be enhanced by incorporating feedback on their EMA responses, during therapy sessions, or with an overview or visualisation of last week, to encourage reflection. This finding resonates with a qualitative study on EMA for veterans with Non‐Suicidal Self‐Injury (NSSI), which revealed interest amongst participants in reviewing their responses to monitor changes and behavioural patterns (Gromatsky et al. 2022). Furthermore, a qualitative study on EMA, which did incorporate feedback, reported generally positive comments regarding this aspect (Bos et al. 2020). These findings highlight the importance for therapists and researchers in blended care to not only use eHealth applications to integrate therapy in daily life, but to also let eHealth bring daily life experiences into therapy. For example, by exploring last week's responses on EMA or by evaluating if and how EMI exercises and metaphors were helpful. This integration ultimately enhances the overall effectiveness of the intervention.

Extending the period of use of the ACT‐DL app, beyond the ACT sessions, was suggested by our participants to prolong the effectiveness of ACT and prevent loss of recently acquired skills. Building upon this idea, ACT‐DL with prolonged or repeated ACT‐DL app use could assist in sustaining and reinforcing the treatment and might serve as secondary prevention. Quickly re‐deploying previously learned skills, like self‐awareness, acceptance, practising mindfulness and committing to one's personal values has the potential to reduce the burden of symptoms and symptom‐related stress. Unfortunately, access to the ACT‐DL app after the study period could not be granted to participants of INTERACT, as the app is awaiting approval as a medical device.

In terms of applicability in daily social‐, study‐, and work‐related routines, participants had doubts about the current design and protocol of the ACT‐DL app. They had various concerns, including lengthy questionnaires, frequent beeps, the repetitive order of questions, inconvenient timing, and a short timeframe for completing questionnaires. The repetitive nature of questions in EMA was mentioned by many participants as negatively influencing the validity of their answers. In other qualitative studies on EMA (Gromatsky et al. 2022; Moitra et al. 2017) a few participants expressed the repetitive nature of questions as negative, but the link with reduced validity of answers has not been made or not been described. Presentation of the questions in random order or using a planned missing‐data design (Silvia et al. 2014), where a selection of the items is presented at each beep, may provide a solution. Our participants proposed an app design that allows customisation, enabling choices such as selecting the number of daily beeps or adjusting response timeframes, or setting the beeps to a lower frequency during work, study, or social situations. In other qualitative EMA studies, similar recommendations were made by participants: to personalise the questionnaires' content, frequency, and duration to better fit participants' situation and treatment goal (Bos et al. 2020) and to allow participants to take a survey at their convenience rather than waiting for a scheduled beep (Moitra et al. 2017). It has been shown that particularly the length of the questionnaire may contribute to a higher burden and more careless responding (Eisele et al. 2022), thus advocating for using shorter questionnaires.

A qualitative study that conducted focus groups with nine individuals who had experienced psychosis, exploring their ideas regarding the potential use of ESM (with EMA and EMI options) for individuals with psychosis (de Thurah et al. 2023), revealed that participants expressed concerns about frequent assessments potentially being burdensome. Additionally, they advised personalising content to increase the potential usefulness of ESM. Thus, even without ever having used ESM, these participants expressed similar ideas on how to make these digital interventions useful and usable for people with (early) psychosis, to our participants. This emphasises the importance of involving the individuals for whom treatments are intended in the earliest stages of development.

A methodological characteristic of the current study that requires further comment is that it was not possible to instal the ACT‐DL app on participants' personal phones. Our interviews revealed that several participants found it very inconvenient to carry and use an additional mobile phone. This concern aligns with findings from other qualitative studies that employed separate devices for EMA (Gromatsky et al. 2022; Moitra et al. 2017). Most of these participants also expressed feelings of shame related to using the study phone in social settings, sometimes noting that it had attracted unwanted attention or ridicule and that it led to reduced adherence. Furthermore, previous research has demonstrated that stigma and self‐stigma in individuals with CHR‐P (Waters et al. 2025) and psychosis (Wood et al. 2017) are related to feelings of shame and are negatively associated with subjective recovery, self‐esteem and social functioning (Vass et al. 2017; Ali et al. 2024). Given these findings, it is crucial that mobile health interventions are made available on users' personal phones. This is particularly important for the typically young individuals with CHR‐P and FEP, to reduce shame and stigma related to their symptoms and to improve treatment adherence.

### 4.3 Limitations

The fact that the INTERACT study was an RCT limited the ACT‐DL protocol in terms of flexibility and customisation. Additionally, as part of the RCT, there was a week of ESM measurements pre‐ and post‐ACT intervention, wherein participants received prompts with 58 questions, ten times a day (Reininghaus et al. 2019). However, during the intervention period they received prompts with 17 questions, 10 times a day for three consecutive days after each session. The study‐related pre‐ and post‐measurements may have significantly biassed the perceived overall burden of the ACT‐DL app.

Considering the trustworthiness criteria (Korstjens and Moser 2018; Nowell et al. 2017), the level of credibility in our study was not optimal, as not all strategies of this criterion were employed. For instance, we only utilised one method of data collection (semi‐structured individual interviews), limiting our method triangulation. Additionally, we did not implement member checks, which involve seeking feedback on data and analysis from participants. Despite these limitations, credibility is reinforced by other strategies such as prolonged engagement, persistent observation, investigator triangulation, and partial data triangulation. Another potential limitation of our study is the use of convenience sampling, which may have introduced biases.

Although our sample was largely heterogeneous and provided a diverse array of perspectives, it exhibited a skewed distribution towards FEP participants (65%) compared to those classified as CHR‐P. This contrasts with the overall INTERACT study population, in which 47% of participants were individuals with FEP. Additionally, a greater number of participants in our sample were employed or enrolled in education (59%), as opposed to being unemployed or on sick leave, whereas the total INTERACT sample included 50% studying or employed individuals. Interestingly, our sample not only included more FEP participants, but also appeared to reflect a higher level of functioning compared to the total INTERACT sample. This may suggest that we included a relatively healthier and/or more educated subgroup of individuals with FEP. It is difficult to determine the exact impact of this on our findings, but it is possible that our sample was better positioned to benefit from the intervention than the average participant in the broader INTERACT population. Our sample also consisted of a relatively large proportion of female participants (71%), which may limit the generalisability of our findings to populations with another gender identity. Finally, the mean age of our sample was 28 years, a little older than the mean age of 25 in the INTERACT sample and the general age of onset of CHR‐P and FEP (16–35), but we do not expect this relatively modest difference has impacted on current results.

## 5 Conclusions

The analysis of 17 semi‐structured interviews with CHR‐P and FEP individuals suggests that integrating the smartphone‐based ACT‐DL app between eight ACT sessions helps embed ACT principles into their daily routines. A majority positively mentioned increased awareness generated by EMA through questionnaires and appreciated the connection between ACT sessions and EMI, particularly the ACT‐ and mindfulness exercises. To further enhance the effects of the ACT‐DL app, participants suggest incorporating feedback on provided answers and placing greater emphasis on the ACT‐ and mindfulness exercises.

Some participants experienced no discernible impact, irritation, or unintended effects (increased contemplation) associated with the ACT‐DL app. Furthermore, challenges arose with the applicability of the ACT‐DL app within the social, academic, or occupational routines of participants. The specific reasons for these challenges were individual and diverse, and no apparent link was found with their early psychotic state. These findings underscore the necessity to develop EMIs that respect individuals' daily activities, privacy preferences, and capabilities. Drawing from the advice of our participants, this means that we need to create apps that are delivered on personal devices, with the flexibility to adjust certain parameters, like notification frequency, input interval after a beep, and overall duration of use.

## Ethics Statement

The qualitative study was an amendment to the “INTERACT trial‐A real‐time and real‐world intervention focusing on stress and reward”. This trial was prospectively registered in the Dutch Trial Register (ID: NTR4252) and was granted ethical approval by the Medical Ethical Committees of Maastricht and Leuven (NL46439.068.16; s59127).

## Consent

All participants provided written informed consent before the interview.

## Conflicts of Interest

The authors declare no conflicts of interest.

## Supporting information


**Data S1:** Supporting Information.

## Data Availability

The data that support the findings of this study are available on request from the corresponding author. The data are not publicly available due to privacy or ethical restrictions.
