# Supplementary material for: Perspectives of Patients With Early Psychosis on the Use of an App in Acceptance and Commitment Therapy: A Qualitative Study
Source: Early Interv Psychiatry. 2025 Aug 5;19(8):e70073. doi: 10.1111/eip.70073 (PMC12323565; doi:10.1111/eip.70073)

Supplement 1. Example of how an ACT Metaphor on Acceptance was explained in a session and workbook.

**Metaphor: Tug-of-War**

The situation you are in right now is a bit like this:

Imagine you are in a tug-of-war with a monster, your pain. It is big, ugly, and very strong. Between you and the monster, there is a huge, gaping hole, that you are slowly being pulled toward. If you lose the tug-of-war, you will fall over the edge and not much will be left of you. So, you pull and pull, trying to avoid that outcome. But the harder you pull, the harder the monster pulls back—and the closer you get to the edge.

In that moment, it is hard to realize that you only need to let go of the rope…


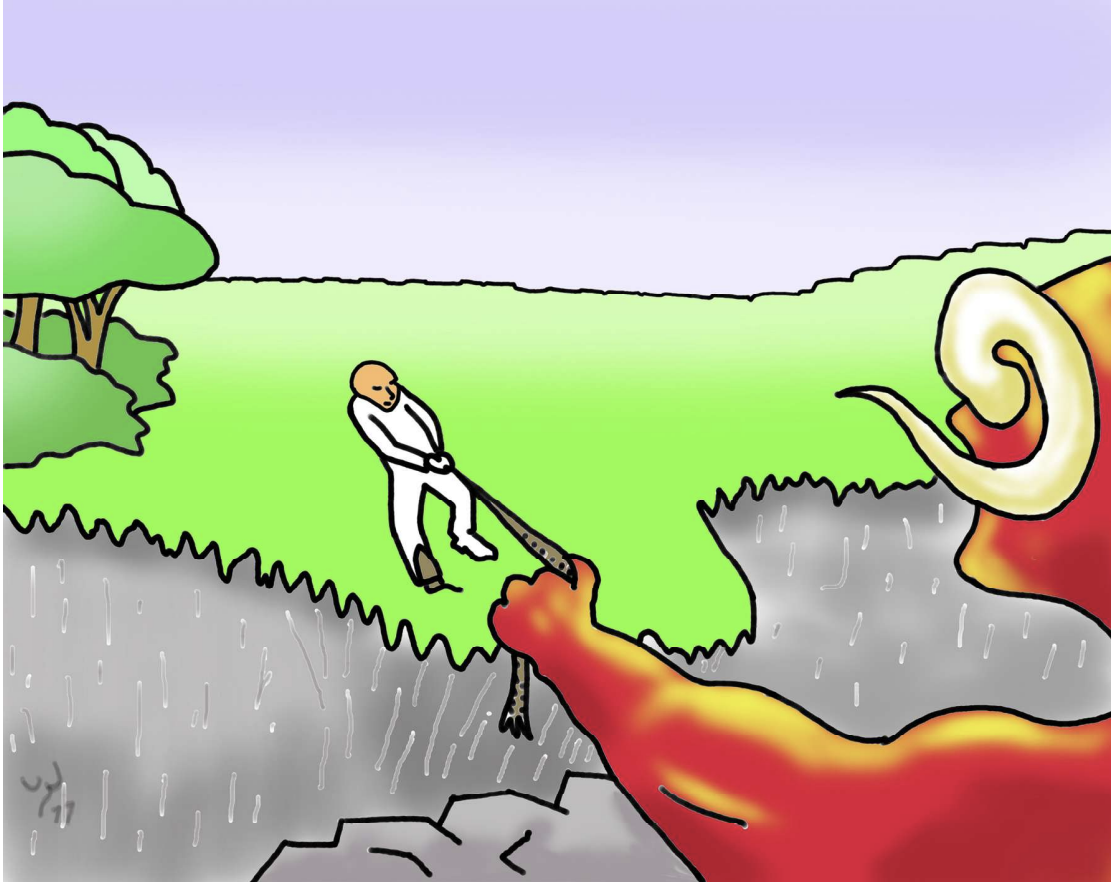

Supplement: Supplementary file 1 — Data S1: Supporting Information. [file EIP-19-0-s001.docx]
